# Hypolipidemic and Antioxidant Activity of Enoki Mushrooms (*Flammulina velutipes*)

**DOI:** 10.1155/2014/352385

**Published:** 2014-08-31

**Authors:** Ming-Yei Yeh, Wen-Ching Ko, Li-Yun Lin

**Affiliations:** ^1^Department of Bioindustry Technology, Dayeh University, Dacun, Changhua 51591, Taiwan; ^2^Department of Food Science and Technology, Hungkuang University, Shalu, Taichung 43302, Taiwan

## Abstract

According to the literatures, *Flammulina velutipes* contains biologically active components such as dietary fiber, polysaccharide, and mycosterol, whose effects in reducing blood sugar, blood pressure, and cholesterol have been proven. This study used the active components extracted from *Flammulina velutipes* powder (FVP) and *Flammulina velutipes* extract (FVE) to investigate the impact of these active components on lipid metabolism of hamsters. The results show that the total dietary fiber content in FVP and FVE is 29.34 mg/100 g and 15.08 mg/100 g, respectively. The total mycosterol content is 46.57 ± 0.37 mg/100 g and 9.01 ± 0.17 mg/100 g, respectively. The male hamsters were subjected to lipid metabolism monitoring by adding 1, 2, and 3% FVP or FVE into their diets for a period of 8 weeks. The animal assay results show that the 3% FVP and FVE groups have the lowest concentration of TC (total cholesterol), TG (triacylglycerol), LDL (low density lipoprotein cholesterol), and LDL/HDL (high density lipoprotein cholesterol) in the serum and liver (*P* < 0.05). Our results demonstrate that the addition of 3% FVP or FVE has a significant effect on the lipid metabolism in hamsters whose increased level of HDL in the serum was induced by high fat diet.

## 1. Introduction

*Flammulina velutipes* is also known as enoki mushroom, golden mushroom,* Basidiomycotina*, Agaricales, Tricholomataceae, and* Flammulina. *It is rich in vitamin B1 and contains traces of zinc. As a dietary supplement,* F. velutipes* is known to be beneficial to people with hypertension, the elderly, and growing children [1]. Its biological activity can help reduce blood sugar, blood pressure, and cholesterol in addition to its antithrombotic effects. It has no known toxic effect on the human body and is very beneficial to human health [2]. Dietary fiber is an active polysaccharide, which is part of the edible portion of* F. velutipes* and cannot be decomposed by lytic enzyme or digested in human alimentary tract. This water-soluble dietary fiber can be combined with cholesterol or cholic acid by adsorption, reducing the amount of cholic acid returned to liver and increasing the metabolism of cholesterol and its transformation into cholic acid. The cholesterol concentration is reduced and the absorption of lipids in the small intestine is disturbed; therefore, the lipid content in blood and the probability of cardiovascular disease can be reduced [3]. The higher the concentration of water-soluble dietary fiber ingested, the better its efficacy in reducing blood cholesterol. It has been shown that feeding mice with 1% water-soluble fiber is sufficient to reduce lipids and triglyceride (TG) in their blood and the level of TG in their liver [4]. It has been reported that water-soluble fiber is significantly more capable of regulating hypolipidemic activity compared with non-water-soluble fiber [5]. Thus, water-soluble dietary fiber is capable of combining bile salts and rendering a hypolipidemic effect [6]. It has been demonstrated that mycosterol can reduce the concentration of total cholesterol and LDL in blood and plasma [7]. The polyphenol compounds in mushrooms are known to reduce the risk of cardiovascular disease and cancer. These polyphenol compounds, such as quercetin, catechin, gallic acid ester, and caffeic acid ester, can prevent the cytotoxicity caused by H_2_O_2_ and the oxidative damage caused by anti-free radicals is also useful in reducing LDL oxidation, DNA damage, and the incidence of cancer [8]. It has also been shown that fruiting body extracts from* F. velutipes* effectively scavenged α-α,diphenyl-picrylhydrazyl (DPPH) free radicals and displayed reducing power [9].

The aim of this study is first to analyze the functionally active components extracted from* F. velutipes* powder (FVP) and* F. velutipes* extracts (FVE). Both FVP and FVE are then added to the diet of hamsters to investigate whether, and to what extent, the active components in* F. velutipes* regulate the metabolism of lipids and antioxidant activity in hamsters with a high fat diet.

## 2. Materials and Methods

### 2.1. Chemicals

Cholesterol, LDL-C, HDL-C, and triglyceride were obtained from ICN Biomedicals, Inc. (Irvine, CA). Bioassay kits, including the triglyceride kit, cholesterol kit, LDL-C kit, and HDL-C kit, are manufactured by Teco Diagnostics (Anaheim, CA). Acetonitrile (LC grade, purity 99%) is provided by Tomowa Chemical Co. (Taichung, Taiwan). Authentic oxalic, citric, malic, α-α,diphenyl-picrylhydrazyl (DPPH), free radicals, linoleic acid, butylated hydroxyanisole (BHA), rutin, quercetin, and decanoic acid are obtained from Sigma Chemical Co. (St. Louis, MO). Ethylenediaminetetraacetic acid (EDTA) is purchased from Mallinckrodt Co. (Hazelwood, MO). Phosphotungstic acid is obtained from J. T. Baker Chemical Co. (Phillipsburg, NJ).

### 2.2. Material

*Flammulina velutipes* used in this study was purchased from Taishen Mushroom located in Taichung, Taiwan. The experimental procedures are as follows.

1. FVP: 300 g of fresh* F. velutipes* was dried with a hot air dryer at 45°C for 48 h and then powdered by a grinding machine for future use.
2. FVE: 300 g of fresh* F. velutipes* was cooked in 600 mL of boiling water for 5 min in 6 batches and then removed. The blanching water was concentrated (3.8 Brix) and freeze-dried for future use.
3. Ingredients of the experimental animal meals are given in Table 1.

### 2.3. Chemical Characterization

#### 2.3.1. Determination of Dietary Fiber

Dietary fiber was analyzed according to the AOAC method [10]. 1 g of the sample is used for the test of insoluble and soluble fibers, respectively.

#### 2.3.2. Determination of Polysaccharide

We used a modified version of the method described by Liu et al. [11]. 3 mg/mL sample solution was prepared with 0.15 M NaCl for gel filtration chromatography; the bed volume of the chromatography column was measured with 5 mg/mL blue dextran when it was confirmed that there was no sugar reaction in the leaching liquor. The 280 nm absorbance of the solution was detected by a UV/VIS detector (UA-6, ISCO, USA) before being collected with a fraction collector (Retriever TM 500, ISCO, USA). Each 2.5 mL was collected in a test tube.

The total sugar of the collected samples was measured by phenol-sulfuric acid coloration, and the protein standard was used to preliminarily measure the molecular weight of the polysaccharide molecules. The chromatographic conditions are described as follows: column: Pharmacia column (1.6 × 90 cm), gel: Sephacryl S-400-HR (Sigma Chemical Co., USA), mobile phase: 0.15 M NaCl, sample concentration: 10 mg/mL, and flow rate: 0.5 mL/mg, 2.5 mL/min.

#### 2.3.3. Determination of Mycosterol

Mycosterol was analyzed by the method described by Feng et al. [12]. 5 g samples were mixed at a 1 : 10 (v/v) ratio with normal hexane for 2 h reflux extraction; this procedure was repeated twice. The residues were then mixed at a 1 : 10 (v/v) ratio with a methanol solvent for 1 h reflux extraction. The filtered solutions were merged and then concentrated by decompression until they were dry. An internal standard (1 mL of 1 mg/mL 5-cholestane) was added and saponified at room temperature for 20 h and was then mixed with 25 mL of chloroform and extracted twice. The lower layer was then taken and mixed with 2 g of anhydrous sodium sulfate to remove water and was then filtered; the filtered solution was concentrated by decompression at 40°C until it was dry. A total of 100 *μ*L of BSTFA + TMCS (99 + 1) was added to the concentrate, and the reaction was allowed to proceed for 2 h at 70°C. The mycosterol content is measured with GC-FID and GC-MS.

### 2.4. Antioxidative Ability Assay

#### 2.4.1. DPPH Radical Scavenging Ability

The radical scavenging ability was analyzed with the method by Shimada et al. [13]. 1 mL sample was mixed with 4 mL of methanol and then uniformly mixed with 1 mL of 0. 2 *μ*M DPPH methanol solution. Samples then rested for 30 min before their absorbance at 517 nm was measured by a spectrophotometer. The formula for calculating scavenging effect is as follows:

(1) Scavenging  effects%=[1−(sample  absorbance  valueabsorbance  value  of  control  group  without  sample)] ×100%.

#### 2.4.2. Ferrous Ion Chelating Ability

Ferrous ion chelation was analyzed according to Dinis et al. [14]. 0.1 mL sample was mixed with 3.7 mL methanol and 0.1 mL of 2 *μ*M FeCl_2_ and waited for 30 seconds before mixing with 0.1 mL of 5 mM ferrozine. The sample rested at room temperature for 10 min and then was collected for 562 nm absorbance test by Hitachi U-2001 spectrophotometer.

The formula for calculating chelating ability is as follows:

(2) Chelating  ability(%)=[1−(sample  absorbance  valueabsorbance  value  of  control  group  without  sample)] ×100%.

#### 2.4.3. Reducing Power

The reducing power was analyzed according to Oyaizu [15]. 0.15 mL samples were mixed with 0.15 mL of 0.2 M pH 6.6 phosphate buffer solution and 0.15 mL of 1% potassium ferricyanide solution, reacted in a water bath at 50°C for 20 min, and cooled in an ice bath for 3 min. Samples were then uniformly mixed with 0.15 mL of a 10% trichloroacetic acid solution and centrifuged and then 0.6 mL of the supernatant was collected and uniformly mixed with 0.6 mL of deionized water and 0.1 mL of 0.1% ferric chloride solution. The mixture reacted in the dark at room temperature for 10 min and then the absorbance at a wavelength of 700 nm was measured by spectrophotometer.

### 2.5. Animal Experiments

All the animal studies performed were approved by the Supervising Animal Ethic Committee of Hungkuang University in accordance with the Helsinki Declaration of 1975.

#### 2.5.1. Animals and Diets

The animal diet is detailed in Table 1. Selected 5-6-week old male Syrian hamsters were used in this study and housed individually in stainless steel cages. The temperature was 24 ± 1°C, the humidity was 40 ~ 60%, and the daily light cycle was 12 h. After one week of ambience adaptation, they were randomly divided into 8 groups of 6 hamsters each. One group of hamsters was given a normal diet (N) and 7 groups of hamsters were given a high fat high cholesterol diet (H). After two weeks of high fat inducement, either 1, 2, and 3% FVP or 1, 2, and 3 % FVE was added to the diet of one of the high fat groups and no addition was made in one high fat group (see Table 1). The experiment lasted for 8 weeks. Animal body weight was recorded daily, whereas the intake of water and food was recorded every two days (Table 5). The animals fasted overnight at the end of the experiment, and samples were collected for analysis, including feces, liver, and blood. The animals were etherized and dissected; the blood samples were extracted from abdominal aorta to analyze total triglyceride (TG), total cholesterol, low density lipoprotein (LDL), and high density lipoprotein (HDL) content. All samples were stored at −80°C so that they were ready for the measurement of the blood lipid.

#### 2.5.2. Determination of Lipoproteins in Serum and Liver

Sera collected from the above were assayed for levels of total cholesterol, low density lipoprotein cholesterol (LDL-C), high density lipoprotein cholesterol (HDL-C), and triglyceride (TG) according to Richmond [16, 17]. Therefore, an enzymatic CHODPAP method with a Teco Diagnostics kit was used for the determination of serum total cholesterol. A cholesterol control reference supplied by the manufacturer was treated with the same manner for calibration. LDL-C was measured spectrophotometrically at 500 nm. On the other hand, the enzyme GDP-PAP triglyceride kit (Teco Diagnostics) was used to determine the serum TG content by following the instructions given by the manufacturer (Lin et al.) [17].

The extraction of hepatic phospholipids contents was carried out according to Folch et al. [18]. The following procedures were described elsewhere by Lin et al. [17]. The method of Bartlett [19] was followed for its determination. The color reaction proceeded with perchloric acid (70%)-ammonium molybdate (2.5%)-ascorbic acid (10%) method of Bartlett [19]. The absorbance was measured at 820 nm. The concentration of hepatic phospholipids was calculated from the calibration curve established using a standard sample supplied by the manufacturer.

### 2.6. Statistics

The experimental results were analyzed by a single factor ANOVA using a computer statistics analysis software SPSS 10.0 (SPSS, Chicago, USA) for intergroup comparisons, and Duncan's multiple range test analysis method was used to determine the significant differences among the various groups (*P* < 0.05).

## 3. Results and Discussion

### 3.1. Dietary Fiber, Polysaccharide, and Mycosterol Content

Dietary fiber is often found in mushroom. It is not digestible by the human alimentary tract, but it increases the solid mass of feces because it is unabsorbed. However, it stimulates gastrointestinal peristalsis and bonds to bile salt, which is then excreted. It can also accelerate the decomposition of cholesterol which reduces its concentration in the blood. The total dietary fiber content in FVP and FVE was 29.34 mg/100 g and 15.08 mg/100 g, respectively (Table 2); the insoluble fiber content was 15.28 mg/100 g and 2.22 mg/100 g, respectively; and the water-soluble fiber content was 14.09 mg/100 g and 12.86 mg/100 g, respectively. The majority of soluble fibers were dissolved by blanching; therefore, the content of soluble fiber in FVE was equivalent to that in FVP. The content of raw fiber and total soluble sugar in* F. velutipes* are higher than in other mushrooms, as shown in the following descending order:* F. velutipes* >* Lentinus edodes* >* Oyster cap fungi* >* Cap fungi *[20]. It is known that, among these mushrooms, the more viscous the water-soluble dietary fiber is, the more significantly it can reduce blood cholesterol [3]. The FVP and FVE polysaccharide content was 15.23 mg/100 g and 20.34 mg/100 g, respectively (Table 2). The fruiting body of FVP had a high content of insoluble fibers. More polysaccharides were found in FVE (20.34 mg/100 g) than in FVP (15.23 mg/100 g) since polysaccharides are water soluble.

In the natural environment, mycosterol mainly exists in a free, esterified, and glycoside form. The chemical structure of mycosterol resembles a fat-soluble substance; its mechanism of action is similar to that of cholesterol in vertebrates [7]. It has been reported that both the spores and the tube tissue in the polypore fungus* G*.* lucidum* have a considerably higher percentage of ergosterol esters than the pileus and stipe tissues [21] because of the presence of the higher amount of crude fiber [22]. It is possible that free ergosterol in the cell membrane of the dead fungal hyphae undergoes either degradation or esterification. The results suggest that free ergosterol (not total ergosterol) should be used as a beach marker for fungal biomass [21]. This experiment used GC/MS for verification and identified 8 types of steroids in addition to the total mycosterol in FVP and FVE. The mycosterol content was 46.57 ± 0.37 mg/100 g and 9.01 ± 0.17 mg/100 g, respectively (Table 3). The total mycosterol content in FVP was more than five times higher than that in FVE, which may be due to the short-term blanching effects on dissolution of mycosterol. The common mycosterol component in mushroom is ergosterol; its content in FVP was 3.02 mg/100 g, but it was not detected in FVE. Stigmastan-3,5-diene was not detected in FVP, but it was 2.85 mg/100 g in FVE; the ergosta-7,22-dienol content was 0.58 mg/100 g in FVP and was 1.94 mg/100 g in FVE.

### 3.2. Antioxidant Activity

From the data shown in Table 4, FVE has a strong antioxidative effect (99.7% DPPH scavenging rate). Yang et al. [20] conducted tests with 6.4 mg/mL methanol extract of assorted edible mushrooms;* Pleurotus geesteranus* can scavenge 81.8% of DPPH free radicals, whereas* Flammulina velutipes, Lentinus edodes,* and* Oyster cap fungus *can scavenge 43~70% of DPPH free radicals. In another test, when the sample concentration is at 1.6 mg/mL,* Flammulina velutipes, Pleurotus geesteranus, Oyster cap fungus,* and* Lentinus edodes* can chelate 45.6~81.6% of ferrous ions. Medicinal mushroom may be used as a source of oxidation-resistant substance and may scavenge free radicals. Phenolic compounds represent the largest group of phytochemical components and phenolic compounds which can inhibit LDL oxidation. The results indicate that FVE can be good antioxidants.

### 3.3. Animal Test

The maximum weight of a male adult hamster can be 160–180 g. Although the hamsters of different groups were fed with different levels of* F. velutipes* daily during the 8-week period, there was no significant influence on the hamster feeding efficiency.

The Syrian hamster is an animal that is widely used in lipid research, as its lipoprotein metabolism and conversion are similar to those of humans [23]. The current study compared 1, 2, and 3% of FVP and FVE groups with the H group to investigate the biochemical values in the blood of hamsters. The hamsters were under monitoring for 8 weeks. As shown in Table 5, the triglyceride (TG) level in the serum of the FVP3 group is 79.00 mg/dL and that in FVE3 group is 85.00 mg/dL, comparing to 119.00 mg/dL in the H group. The total cholesterol in the FVP3 group is 259.00 mg/dL and that in FVE3 group is 265.00 mg/dL, comparing to 363.00 mg/dL in the H group. The LDL-C in the H group is 132.00 mg/dL which is much higher than 60.00 mg/dL in the FVP3 group and 68.00 mg/dL in the FVE3 group. The HDL-C in the H group is 174.00 mg/dL which is much lower than 204.00 mg/dL in the FVP3 group and 211.00 mg/dL in the FVE3 group. We found that both ratios of LDL-C/HDL-C and TC/HDL-C are lower in the groups with higher dosage (up to 3%) of FVP and FVE. These high FVP/FVE content groups show an obvious trend in lower TG in serum, and the LDL/HDL ratios are lower in FVP groups than in FVE groups. Also, higher FVP and FVE contents have an impact on decreasing the TC/HDL ratio (Table 6).

With regards to triglyceride in the liver, TG is 51.90 mg/dL in the H group which is much higher than 28.00 mg/dL in the FVP3 group and 31.00 mg/dL in the FVE3 group. The total liver cholesterol in the H group is 45.70 mg/dL which is much higher than 28.50 mg/dL in the FVP3 group and 23.10 mg/dL in the FVE3 group. Also, as shown in Table 5, the liver phospholipid in the H group is 14.10 mg/dL, compared to 17.30 mg/dL in the FVP3 group and 20.30 mg/dL in the FVE3 group. As shown in Table 2, the soluble fiber in FVP and FVE was 14.09 mg/100 g and 12.86 mg/100 g, respectively. Previous study indicates that adding 1% of soluble fiber to the daily diet of mice can reduce blood lipid and liver TC and TG concentrations significantly. A higher concentration of soluble dietary fiber is more effective in reducing blood cholesterol [5] FVP and FVE reduce the TG and TC concentration in the liver, and FVP has stronger effect than FVE (Table 6). Studies also indicate that higher phospholipid content is more helpful in reducing the total cholesterol level in liver [24]. In Table 3, the total mycosterol contents in FVP and FVE are 46.57 and 9.01 mg/100 g, respectively, which can reduce blood sugar, blood pressure, and cholesterol and resist thrombosis.

According to Ostlund Jr., [25] an average person ingests only 0.15~0.45 g of mycosterol in his or her daily diet; therefore, in order to benefit from mycosterol for cholesterol reduction, additional supplementary mycosterol is required in the daily diet. Large amounts of meat and fat are ingested in our daily diet; if sufficient mycosterol is contained in the diet, it would compete with cholesterol in the intestinal tract so less cholesterol can be absorbed by body and the blood cholesterol could be reduced.

## 4. Conclusion

1. *Flammulina velutipes* is rich in polysaccharides and can be used in health food and cosmetic products for its biological activity.
2. The antioxidant activity test shows that FVE is displayed to be as high as 99.7%, which could prevent free radical and oxygen attacks. It also showed a metal chelating ability.
3. *F. velutipes* contains rich dietary fiber, which accelerates the decomposition of cholesterol and hence can reduce TG, TC, and LDL level in the blood and TG, TC, and phospholipid in the liver.
4. Mycosterol has the ability to reduce total cholesterol and LDL in the blood. Taiwan has a high yield of* Flammulina velutipes* all year round which has been developed into nutritional supplementary foods, and it is becoming an attractive research subject in the 21st century.

## Figures and Tables

**Table 1:** Ingredients of the experimental animal meals (%)^a^.

Ingredients	Group							
	N	H	FVP1	FVP2	FVP3	FVE1	FVE2	FVE3
Casein	20.0	20.0	20.0	20.0	20.0	20.0	20.0	20.0
Sucrose	15.0	15.0	15.0	15.0	15.0	15.0	15.0	15.0
Corn starch	50.0	45.0	45.0	45.0	45.0	45.0	45.0	45.0
Corn oil	2.5	5.0	5.0	5.0	5.0	5.0	5.0	5.0
Lard	2.5	5.0	5.0	5.0	5.0	5.0	5.0	5.0
Mineral	3.5	3.5	3.5	3.5	3.5	3.5	3.5	3.5
Vitamin	1.0	1.0	1.0	1.0	1.0	1.0	1.0	1.0
Choline	0.2	0.2	0.2	0.2	0.2	0.2	0.2	0.2
Methionine	0.3	0.3	0.3	0.3	0.3	0.3	0.3	0.3
α-Cellulose	5.0	5.0	5.0	5.0	5.0	5.0	5.0	5.0

Total	100	100	100	100	100	100	100	100

^a^On the basis of AIN-76 formula (American Institute of Nutrition (AIN)).

Amounts of corn oil, buckwheat seeds, and sprouts added were based on percent weight. N: normal diet; H: high fat diet; FVP1, FVP2, and FVP3: feed with 1%, 2%, and 3% FVP; FVE1, FVE2, and FVE3: feed with 1%, 2%, and 3% FVE.

**Table 2:** Contents of dietary fiber and polysaccharide of FVP and FVE.

Components	Contents (mg/100 g)	
	FVP	FVE
Total dietary fiber	29.34 ± 0.72	15.08 ± 0.26
Insoluble dietary fiber	15.28 ± 2.22	2.22 ± 0.26
Soluble dietary fiber	14.09 ± 2.94	12.86 ± 0.52
Polysaccharides	15.23 ± 2.22	20.34 ± 0.21

Reported values are the mean ± standard deviation (S.D.) (*n* = 3).

**Table 3:** Mycosterol composition of FVP and FVE.

Components	Contents (mg/100 g)	
	FVP	FVE
Stigmastan-3,5-diene	ND	2.85 ± 0.09
Ergosterol	3.02 ± 0.02	ND
Ergosta-4,6,22-trienol	3.04 ± 0.02	ND
Ergosta-7,22-dienol	0.58 ± 0.01	1.94 ± 0.02
Ergosta-4,22-dienone	2.41 ± 0.03	ND
Ergosta-5,7-dienol	0.87 ± 0.01	ND
Ergosta-8,14-dienol	10.71 ± 0.19	ND
Fungisterol	2.51 ± 0.08	ND
Other	23.43 ± 0.49	4.22 ± 0.03
Total mycosterol	46.57 ± 0.37	9.01 ± 0.17

ND: not detected.

**Table 4:** Antioxidant activities of FVP and FVE.

Items	1 mg/mL	
	FVP	FVE
DPPH scavenging %	21.6 ± 0.19	99.7 ± 3.62
Chelating ability of ferrous %	10.9 ± 0.01	46.4 ± 0.07
FRAP reducing power %	17.1 ± 0.19	68.1 ± 1.07
Total phenols (ppm)	164.5 ± 6.19	4598.0 ± 2.22

Reported values are the mean ± standard deviation (S.D.) (*n* = 3).

**Table 5:** Effect of dried FVP and FVE on food intake, body weight, and feed efficiency of hamster^a^.

Grow the parameters	N	H	FVP1	FVP2	FVP3	FVE1	FVE2	FVE3
Food intake	7.56 ± 0.12	7.38 ± 0.10	7.74 ± 0.15	7.81 ± 0.22	7.52 ± 0.11	7.64 ± 0.17	7.57 ± 0.18	7.58 ± 0.15
Body weight (g)	80.67 ± 1.53	105.83 ± 8.20	98.62 ± 3.26	103.00 ± 1.95	105.00 ± 2.35	101.00 ± 2.38	103.00 ± 2.24	109.00 ± 3.05
Feed efficiency	1.58 ± 0.02	1.54 ± 0.03	1.59 ± 0.03	1.57 ± 0.03	1.64 ± 0.03	1.59 ± 0.02	1.56 ± 0.04	1.56 ± 0.02

^a^N: normal formula; H: high lipid diet; FVP1, FVP2, and FVP3: feed with 1%, 2%, and 3% FVP; FVE1, FVE2, and FVE3: feed with 1%, 2%, and 3% FVE. Feed efficiency = [wt gain (g)/total diet intake (g)] × 100%.

**Table 6:** Effects of dried FVP and FVE on the nutritional and biochemical parameters related to serum and hepatic levels in male hamsters^a^.

Grow the parameters	N	H	FVP1	FVP2	FVP3	FVE1	FVE2	FVE3
Blood								
TG	62.00 ± 11.90^e^	119.00 ± 12.00^b^	100.00 ± 12.10^bc^	96.00 ± 12.40^cd^	79.00 ± 10.30^de^	110.00 ± 11.20^bc^	132.00 ± 9.80^a^	85.00 ± 1.03^cd^
TC	141.00 ± 9.00^e^	363.00 ± 9.80^a^	287.00 ± 6.70^b^	263.00 ± 8.90^cd^	259.00 ± 9.70^d^	289.00 ± 11.20^b^	309.00 ± 9.80^bc^	265.00 ± 7.90^b^
LDL-C	38.00 ± 4.40^d^	132.00 ± 4.30^a^	59.00 ± 12.40^c^	44.00 ± 9.60^cd^	60.00 ± 8.80^cd^	106.00 ± 6.20^b^	125.00 ± 8.30^b^	68.00 ± 10.10^c^
HDL-C	117.00 ± 8.90^e^	174.00 ± 4.40^c^	237.00 ± 6.10^a^	222.00 ± 4.30^abc^	204.00 ± 6.30^bc^	193.00 ± 5.70^bc^	223.00 ± 9.80^abc^	211.00 ± 5.40^abc^
LDL/HDL-C	0.32 ± 0.17^c^	0.76 ± 0.01^a^	0.25 ± 0.02^c^	0.20 ± 0.14^c^	0.29 ± 0.18^c^	0.55 ± 0.13^ab^	0.56 ± 0.15^c^	0.32 ± 0.08^b^
TC/HDL-C	1.20 ± 0.12^c^	2.09 ± 0.02^a^	1.21 ± 0.06^c^	1.18 ± 0.11^c^	1.27 ± 0.06^c^	1.50 ± 0.08^ab^	1.38 ± 0.11^ c^	1.26 ± 0.02^bc^

Liver (mg/g)								
TG	17.70 ± 4.10^a^	51.90 ± 12.40^a^	34.30 ± 16.10^b^	29.60 ± 5.30^b^	28.00 ± 2.90^bc^	39.80 ± 13.20^b^	35.40 ± 1.80^b^	31.00 ± 1.20^b^
TC	14.10 ± 3.70^e^	45.70 ± 4.70^a^	43.10 ± 6.60^a^	28.50 ± 12.80^bc^	28.50 ± 7.40^bc^	44.90 ± 1.40^e^	35.30 ± 7.10^cd^	23.10 ± 0.80^cd^
Phospholipid	7.60 ± 2.10	14.10 ± 1.60	7.80 ± 1.50	13.20 ± 6.10	17.30 ± 2.60	21.80 ± 9.80	21.10 ± 2.20	20.30 ± 2.80

^a^N: normal formula; H: high lipid diet; TC: total cholesterol; TG: triacylglycerol; LDL-C: low density lipoprotein cholesterol; HDL-C: high density lipoprotein cholesterol; LDL/HDL-C = low density lipoprotein/high density lipoprotein cholesterol; TC/HDL-C = total cholesterol/high density lipoprotein cholesterol.

a–e in the same row with different superscripts are significantly different (*P* < 0.05).
